# A survey of regulatory recommendations for waivers of *in vivo* bioequivalence studies of generic products for certain dosage forms by participating regulators and organisations of the International Pharmaceutical Regulators Programme. Part 2

**DOI:** 10.3389/jpps.2025.14721

**Published:** 2025-10-20

**Authors:** Alfredo García-Arieta, Andrew Tam, Florencia Tiseyra, Eduardo Agostinho Freitas Fernandes, Kelen Carine Costa Soares, Raphael Sanches Pereira, Henrike Potthast, Katalina Mettke, Ya-Wen Chang, Li-Feng Hsu, Yu-Lin Su, Zulema Rodríguez Martínez, Milly Divinsky, Kevin Blake, April C. Braddy, Clare Rodrigues, Tiong Toh, Erwin Guzman Aurela, Liliana Carolina Arévalo González, Ben Jones, So Hee Kim, Choongyul Ahn, Eunju Yun, Ryosuke Kuribayashi, Kohei Shimojo, Miho Kasuga, Junya Makino, Joel Raffel, Joy van Oudtshoorn, Philda Tabane, Bader Alzenaidy, Mohammed AlHarbi, Adel Alharf, Albatool BinAjlan, Chantal Walther, Matthias S. Roost, Christopher Crane, Kitty Lee

**Affiliations:** ^1^ WHO-Observer, Geneva, Switzerland; ^2^ Health Canada, Ottawa, ON, Canada; ^3^ ANMAT, Buenos Aires, Argentina; ^4^ ANVISA, Brasília, Brazil; ^5^ BfArM, Bonn, Germany; ^6^ CDE, Taipei, Taiwan; ^7^ COFEPRIS, México, Mexico; ^8^ MOH, Jerusalem, Israel; ^9^ EC/EMA, Amsterdam, Netherlands; ^10^ FDA, Silver Spring, MD, United States; ^11^ HSA, Singapore, Singapore; ^12^ INVIMA, Bogota, Colombia; ^13^ Medsafe, Wellington, New Zealand; ^14^ MFDS, Cheongju-si, Republic of Korea; ^15^ PMDA, Tokyo, Japan; ^16^ MHRA, London, United Kingdom; ^17^ SAHPRA, Pretoria, South Africa; ^18^ SFDA, Riyadh, Saudi Arabia; ^19^ Swissmedic, Bern, Switzerland; ^20^ TGA, Canberra, ACT, Australia

**Keywords:** bioequivalence, biowaivers, regulatory requirements, dosage forms, IPRP BEWGG

## Abstract

A biowaiver generally refers to the request to waive an *in vivo* bioequivalence study. A biowaiver may be granted not only based on the Biopharmaceutics Classifications System (BCS) but also for many immediate-release dosage forms based on pre-defined criteria. The current paper summarises the results from a survey of the biowaiver requirements for cutaneous/topical products (topical solutions, gels, suspensions, ointments, and creams), ear/otic and ophthalmic solutions and suspensions, enemas in solution and suspension, and vaginal solid dosage forms and suppositories defined by the participants of the Bioequivalence Working Group for Generics (BEWGG) of the International Pharmaceutical Regulators Programme (IPRP). A review of the results from the survey indicates that there is a trend towards convergence when the dosage forms are less complex; however, the most common approach used by each of the participants was a case-by-case approach given that most participants do not have well-defined guidelines to support all possible scenarios. Notwithstanding the differences, disseminating information is the first step towards regulatory convergence regarding biowaivers for certain dosage forms and will be useful for pharmaceutical companies currently developing generic medicinal products for countries represented by IPRP participants.

## Introduction

Medicine regulatory authorities are responsible for the assessment and approval of both innovator and generic products, while ensuring that the generic products meet bioequivalence standards with the corresponding comparator product to enhance access to medicines worldwide and affordability.

The International Generic Drug Regulators Programme (IGDRP) was created to promote collaboration and convergence among generic drug regulators to address the challenges posed by the increasing workloads, globalisation, and complexity of scientific issues [1]. In 2018, the IGDRP merged with the International Pharmaceutical Regulators Forum (IPRF) to form the International Pharmaceutical Regulators Programme (IPRP). The IPRP allows its members and observers to exchange information on issues of mutual interest, promote cooperation, maximise synergies, and avoid duplication of effort. It also creates a regulatory hub for manufacturers of all medicinal products and enables linkages with other initiatives to simplify the numerous forms of international regulatory collaboration [1].

The Bioequivalence Working Group for Generics (BEWGG) of IPRP aims to promote greater collaboration, regulatory convergence, and potential mutual reliance on respective bioequivalence (BE) assessments in the longer term. This group is composed of the following regulators/organisations: ANMAT, Argentina; ANVISA, Brazil; COFEPRIS, Mexico; EC, Europe; Health Canada, Canada; HSA, Singapore; INVIMA, Colombia; Medsafe, New Zealand; SAHPRA, South Africa; MFDS, Republic of Korea; MOH, Israel; PMDA, Japan; Swissmedic, Switzerland; TFDA, Chinese Taipei; TGA, Australia; FDA, United States; SFDA, Saudi Arabia; MHRA, the United Kingdom; and WHO, as an observer.

The recommendations to waive *in vivo* BE studies for immediate release of solid oral dosage forms based on the Biopharmaceutics Classifications System (BCS) in the BEWGG of IPRP participants were described previously [2] and have now been harmonised by the International Council for Harmonisation of Technical Requirements for Pharmaceuticals for Human Use (ICH) [3]. Additional work from the BEWGG includes a summary of the expectations for biowaivers for additional strengths of immediate release and modified release solid oral dosage forms [4, 5], which have also been identified as ICH topics for harmonisation. More recently, the BEWGG has published the biowaiver recommendations for systemically acting oral dosage forms including oral solutions, oral suspensions and soft gelatine capsules, and systemically acting injectable products including intravenous, subcutaneous, and intramuscular injections, emulsions for injection, and micellar solutions for injection [6]. This marked the first step towards regulatory convergence on the topic of dosage form biowaivers.

The objective of the current review paper is to describe the biowaiver recommendations and requirements for the following dosage forms among the BEWGG member organizations of the IPRP: cutaneous/topical products (topical solutions, gels, suspensions, ointments, creams, and lotions), ear/otic and ophthalmic solutions, suspensions and ointments, enemas in solution and suspension, and vaginal solid dosage forms and suppositories. As many BEWGG member organizations do not have published guidelines on many of these dosage forms, the sharing of this information is important for facilitating regulatory convergence in this area.

## Materials and methods

The IPRP BEWGG conducted a survey and technical discussions in 2023-2024 on the recommendations and requirements to demonstrate BE for different types of immediate release dosage forms: vaginal solid dosage forms, suppositories, enemas, ear/otic and ophthalmic solutions, suspensions and ointments, and cutaneous/topical products.

This information was obtained from the participating regulatory authorities and organizations in the BEWGG and is based on their respective regulatory guidance documents and policies [7–36].

### Terminology

To some, BE studies refer only to pharmacokinetic (PK) studies; however, for the purpose of the survey, *in vivo* BE studies refer not only to PK studies but also to therapeutic equivalence studies with pharmacodynamic (PD) (e.g., blanching studies) or clinical endpoints or local bioavailability studies such as tape stripping studies. For the FDA, United States, the term “biowaiver” refers to either the decision to waive an *in vivo* BE requirement under 21 CFR 320.22 or the decision to accept *in vitro* BE data in accordance with 21 CFR 320.24(a).

For the purposes of this paper, the term “Q1” refers to the same qualitative excipient composition; however, the acceptability of some excipients such as colorants, fragrances, and preservatives under “Q1” may differ among the varying regulators. The term “Q2” refers to similar quantitative excipient composition, where the range of similarity may be defined in each agency’s guidance documents. The term “Q3” refers to having a similar microstructure based on the similarity of physicochemical properties, which may also be defined in each agency’s guidance documents.

## Results

### Topical products

Topical products are available in several dosage forms: solutions, suspensions, gels, ointments, and emulsions.

PMDA, Japan requires dermatopharmacokinetic studies for all topical dosage forms, including topical solutions [7, 8]; therefore, the demonstration of equivalence with *in vitro* data alone (i.e., biowaivers) is only acceptable for bactericides, disinfectants, and antiseptics.

In Brazil, biowaivers are generally accepted for locally acting drug products not intended for systemic effects. With the exception of semisolid corticosteroids for dermatological use, the topical products must be pharmaceutically equivalent to the comparator product, have the same excipients in the same quantities (i.e., Q1 and Q2), have the same physicochemical and microstructural behaviour, and have comparable *in vitro* release test (IVRT) results [9]. In case of differences in excipients, biowaivers may be accepted on a case-by-case basis if *in vitro* permeation is shown to be similar.

Biowaivers are generally acceptable for locally acting drug products in Argentina [10] and Mexico [11], and any excipient can be changed for another with the same function as that of the comparator product if they are well-established for that pharmaceutical dosage form. For ANMAT, Argentina, the physicochemical properties of the excipients should also be similar. In Australia and Singapore, biopharmaceutic data is not required for dermal products if the drugs are not acting systemically [12, 13], but a PK study may be required for systemic safety if systemic exposure is measurable. In addition, for locally acting products in Australia, a case-by-case application may be made based on Q1, Q2, and justified Q3 data, where the *in vitro* tests are justified as correlating with local levels at the site of action (e.g., single-phase aqueous product). In New Zealand, comparative physical and therapeutic equivalence studies with PD endpoints are not required if the medicine has no systemic action [14].

MOH, Israel accepts biowaivers for topical antibiotics based on the inhibition zone and *in vitro* permeation tests, but biowaivers are not accepted for topical steroids because a PD comparison based on blanching is required. For other topical products, if there is a possibility for systemic adverse reactions, a biowaiver may be possible based on *in vitro* permeations tests (IVPT) [15]. This implies that for topical products not intended for systemic action and without expected systemic adverse reactions, the waiver may be possible on a case-by-case basis without any *in vitro* testing if excipients are well-established for that dosage form.

The WHO guideline defines the requirements for biowaivers for topical aqueous and oily solutions and gels in solution but not for emulsions or suspensions [16]. Topical aqueous and oily solutions and gels in solution should contain the same excipients in similar concentrations. Gels in solution also require comparable IVRT data. The WHO guideline is also followed by INVIMA, Colombia [17].

SAHPRA, South Africa follows a similar approach as the WHO for topical solutions. Topical solutions with bacteriostatic, bactericidal, antiseptic, and/or antifungal claims may qualify for a waiver based on appropriate validated *in vitro* test methods (e.g., microbial growth inhibition zones). For other topical formulations, clinical data (e.g., comparative clinical efficacy) will be required for demonstration of therapeutic equivalence (TE). Proof of release by membrane diffusion will not be accepted as proof of efficacy unless data are presented demonstrating a correlation between release through a membrane and clinical efficacy [18]. In addition, SAHPRA, South Africa is open to alternative approaches if accepted by EC Europe; FDA, United States; or the WHO.

In Switzerland, TE clinical trials are generally required to support the approval of generic topical products given that a guideline describing biowaiver requirements does not exist [19]. Notwithstanding the absence of a guideline, biowaivers may be accepted on a case-by-case basis depending on the dosage form and if certain conditions are met with respect to excipient composition, physicochemical properties, and acceptable scientific justifications.

SFDA, Saudi Arabia and FDA, United States have issued multiple product-specific BE guidelines for several topical products [20, 21] and the methodology employed for concluding similar microstructure based on the physicochemical properties of the product. IVRT and IVPT has also been published by the FDA, United States [22–24].

The EU guideline on the investigation of BE [26] is adopted by TGA, Australia; Medsafe, New Zealand; SFDA, Saudi Arabia; SAHPRA, South Africa; Swissmedic, Switzerland; and MHRA, UK such that BE studies are required whenever the action of a locally applied product depends on systemic exposure [26]. Furthermore, whenever the local application of a locally acting medicinal product, including topical products, entails a risk of systemic adverse reactions, systemic exposure should be measured to ensure that the systemic exposure of the test product is not higher than the exposure of the comparator product (i.e., the upper limit of the 90% confidence interval should not exceed the upper BE acceptance limit 125.00%). These requirements were established when waivers for most topical dosage forms other than solutions were not possible and clinical endpoints were required but could not address the systemic safety profile. HSA, Singapore refers to the ASEAN guideline for the conduct of bioequivalence studies [27], which is adopted from the EU guideline.

Currently, the EU requirements for biowaiver of topical products is in draft format [25] and describes the acceptance criteria for qualitative and quantitative differences between a generic topical product and its comparator product such that the differences should not affect local availability or physicochemical properties.

In Chinese Taipei, biowaivers can be granted when topical products are listed in the OTC monograph [28] or for topical solutions if excipients have Q1, Q2, and Q3 similarity [29]. For other topical products, *in vivo* demonstration of bioequivalence is required. In addition to a clinical study (e.g., PK bioequivalence or therapeutic equivalence based on clinical endpoints), other scientific approaches to establish bioequivalence are also considered acceptable [30]. These include the possibility of using IVRT and, when necessary, IVPT in those cases where the excipient composition and physicochemical properties are sufficiently similar. Similarly, in the Republic of Korea, topical solutions may be exempted from BE demonstration if the API is the same as in the comparator product and excipients do not affect the absorption of active ingredients [31]. For other topical dosage forms, *in vivo* demonstration of BE may be exempted only if the excipients, excluding preservatives, antioxidants, colorants, and flavouring agents, are the same composition (Q1) as the existing comparator product. In case of differences, it should be proven that the excipients do not affect safety and effectiveness through dermatopharmacokinetic, PD, or clinical studies [31]). In these cases, the *in vivo* BE demonstration may be replaced with physicochemical equivalence test data (physicochemical properties considering the dosage form (e.g., pH, specific gravity or density, osmotic pressure, viscosity, etc.)) [32].

Apart from ANMAT, Argentina and COFEPRIS, Mexico, who accept qualitative and quantitative differences in excipients, and MFDS, Republic of Korea, who may accept quantitative differences, biowaivers for the remaining participants can be granted only when the excipient composition of Q1 and Q2 is similar. Minor qualitative and quantitative differences in non-functional excipients that do not affect absorption would be accepted, though there are no specific guidelines. None of the participants have presently defined threshold limits for quantitative differences, except Health Canada, Canada [33]; EC, Europe [25]; TFDA, Chinese Taipei [29]; and FDA, United States in some product specific guidelines (e.g., diclofenac sodium topical solutions [34, 35]). In the USA, a topical test product should contain no difference in inactive ingredients or in other aspects of the formulation to the Reference Standard (RS) that may significantly affect the local or systemic availability. A threshold of ±5% for allowable quantitative differences in inactive ingredients is set for parenterals, otics, and ophthalmic products [36–38]. Topical products may be advised to follow the same criteria as outlined in the product-specific guidelines (e.g., acyclovir cream [39]).

Health Canada, Canada, in principle, may accept qualitative differences on a case-by-case basis (e.g., buffers, preservatives, and organoleptics), as long as these differences are not deemed clinically relevant and do not affect absorption, and quantitative differences of only ± 10%, unless otherwise justified [33]. In the EU, the draft guideline on quality and equivalence of topical products has proposed that those excipients whose function is to influence the active substance solubility, thermodynamic activity or bioavailability, and product performance should be qualitatively the same [25]. The nominal quantitative composition of the excipients should be the same or not differ by greater than ± 5%. For example, for an excipient present in the comparator product at 2%w/w, the permitted range in the test product would be 1.9 – 2.1%w/w. Qualitative differences are permitted for excipients whose primary function is not related to product performance or administration (i.e., antioxidants, antimicrobial preservatives, or colours), that do not have any other functions or effect that influences the active substance solubility, thermodynamic activity or bioavailability, and product performance, that have no effect on local tolerance or safety, and that are substituted by well-established excipients in usual amounts and whose function relates to the vehicle or emolliency (e.g., paraffin homologues). Regarding quantitative difference, a difference of ± 10% is acceptable for excipients whose function only relates to the vehicle properties or emolliency [25]. Similar requirements are employed by TFDA, Chinese Taipei [29] and MOH, Israel [15]. These quantitative limits are applied for all the other topical dosage forms.

Taking into account the general requirements described above for each participant, the following subsections provide additional information that may be required to support a biowaiver for specific topical products.

#### Topical solutions

All participants, except PMDA, Japan, accept biowaivers of *in vivo* BE studies for locally acting topical solutions. Most countries evaluate qualitative and quantitative differences on a case-by-case basis. The physicochemical properties that may be required for topical solutions are appearance, viscosity, specific gravity, drying rate, and/or surface tension [33, 40].

#### Topical suspensions

PMDA, Japan does not consider biowaivers for topical suspensions. MOH Israel consider biowaivers for topical antibiotics based on the inhibition zone and *in vitro* permeation tests. For other topical products that do not contain corticosteroids and for which there is a possibility of systemic adverse reactions, a biowaiver may be possible based on *in vitro* permeation tests [15]. This implies that for topical products not intended for systemic action and without expected systemic adverse reactions, a waiver may be possible on a case-by-case basis without any *in vitro* testing if excipients are well-established for that dosage form.

FDA, United States and SFDA, Saudi Arabia follow a case-by-case approach according to the corresponding product-specific guidance (e.g., Spinosad [20] or betamethasone dipropionate and calcipotriene [41]). ANMAT, Argentina grants biowaivers based on using well-known excipients. In contrast, TGA, Australia; ANVISA, Brazil; Health Canada, Canada; INVIMA, Colombia; EC, Europe; Medsafe, New Zealand; MFDS, Republic of Korea; SAHPRA, South Africa; Swissmedic, Switzerland; TFDA, Chinese Taipei; MHRA, UK; and the WHO may grant a biowaiver based on Q1 and Q2 similarity as well as similar physicochemical properties (Q3). The physicochemical properties that may require comparative assessment include appearance, texture, crystallographic structure, pH, viscosity, micrographs, osmolality, particle size distribution, rheological parameters (shear stress vs. shear rate, viscosity vs. shear rate, apparent viscosity at low, medium, and high shear rates, complete flow rate across the range of attainable shear rates until low or high shear plateaus, and yield stress), water activity, specific gravity or density, and *in vitro* drug dissolution/release. Canada would also require that the gel vehicle is single-phase and aqueous-based. COFEPRIS, Mexico grants biowaivers on a case-by-case approach.

#### Topical gels

PMDA, Japan does not consider biowaivers for topical gels while FDA, United States and SFDA, Saudi Arabia follow a case-by-case approach according to their respective corresponding product-specific guidance (e.g., erythromycin [42]). For example, the FDA, United States has identified a simpler *in vitro* approach compared to other gels (e.g., mechlorethamine hydrochloride [43]) because there is no known or suspected bioavailability problem with the drug product. ANMAT, Argentina and COFEPRIS, Mexico grant biowaivers without a comparison to the comparator product and TGA, Australia; ANVISA, Brazil; Health Canada, Canada; HSA, Singapore; INVIMA, Colombia; EC, Europe; MOH, Israel; Medsafe, New Zealand; MFDS, Republic of Korea; SFDA, Saudi Arabia; SAHPRA, South Africa; Swissmedic, Switzerland; TFDA, Chinese Taipei; MHRA, UK; and the WHO may accept BE to be demonstrated via *in vitro* studies (e.g., justified physicochemical properties and IVRT) if the active substance is in solution. In Canada, IVPT may also be required on a case-by-case basis.

In the case of topical gels with systemic action (e.g., testosterone) a waiver could be possible in Australia, Brazil, Canada, the EU, Israel, Mexico, New Zealand, Saudi Arabia, Singapore, South Africa, Switzerland, Chinese Taipei, the UK and from the WHO based on a comparison of Q1, Q2, and Q3 parameters. Q3 comparisons would be based on physicochemical equivalence of justified parameters and similar *in vitro* release. Although the EMA Guideline on the investigation of bioequivalence [26] indicates that *in vivo* bioequivalence studies for systemically acting products or non-superior systemic exposure for locally acting products with some level of systemic exposure are required, a biowaiver based on the requirements similar to those for a biowaiver for oral solutions could be granted. In case of significant qualitative or quantitative differences, an *in vivo* BE study would be required. For example, the WHO guideline specifies that for non-oral, non-parenteral pharmaceutical products designed to act systemically, such as testosterone gel, *in vivo* studies are necessary, but for pharmaceutically equivalent topical gel products, equivalence can be demonstrated by means of *in vitro* membrane diffusion studies when the products contain essentially the same excipients in comparable concentrations and the API(s) in the product are in solution [16].

In Australia, Brazil, Canada, the EU, New Zealand, Republic of Korea, Singapore, South Africa, Switzerland, Chinese Taipei, and the UK, although not stated in the guidelines, a waiver would be also acceptable for gels where the drug is in suspension with the same requirements as for gels where the drug is in solution. This possibility is not included in the WHO and INVIMA, Colombia’s guidelines. INVIMA, Colombia accepts biowaivers in line with the requirements for gels where the drug is in solution.

The physicochemical properties to compare the products may include appearance, texture, crystallographic structure, pH, drying rate, viscosity, micrographs, osmolality, particle size distribution, rheological parameters (shear stress vs. shear rate, viscosity vs. shear rate, apparent viscosity at low, medium, and high shear rates, complete flow rate across the range of attainable shear rates until low or high shear plateaus, yield stress, and linear viscoelastic response), water activity, surface tension, specific gravity or density, and IVRT. TGA, Australia; Health Canada, Canada; Medsafe, New Zealand; SFDA, Saudi Arabia; MHRA, UK and FDA, United States may require IVPT on a case-by-case basis.

#### Topical ointments

The biowaiver requirements for topical ointments are similar to those of topical gels as described in the previous subsection, but they are not defined in the guidelines from WHO and Colombia; however, Colombia accepts biowaivers in line with the requirements for gels.

The physicochemical properties to compare the products are similar to those of gels and include globule size and the characterization of oleaginous components but not pH, drying rate, osmolality, water activity, or surface tension. TGA, Australia,; Health Canada, Canada; Medsafe, New Zealand; MFDS, Republic of Korea; SFDA, Saudi Arabia; and FDA, United States may require IVPT.

#### Topical emulsions: creams and lotions

Japan does not consider biowaivers for topical emulsions. FDA, United States and SFDA, Saudi Arabia follow a case-by-case approach according to the corresponding product-specific guidance in which some topical emulsions may be waived based only on physicochemical characterisation [25] if excipient composition is sufficiently similar (e.g., fluocinolone acetonide [44]), whereas in other cases more tests are required, including IVRT and IVPT (e.g., acyclovir cream [39] and clindamycin lotion [45]). The remaining participants consider biowaivers of *in vivo* BE studies for topical emulsions in the absence of systemic action on a case-by-case basis. Due to the complexity of emulsions, an *ex vivo* “*in vitro* permeation test” (IVPT) could be an alternative to *in vivo* studies if the qualitative and quantitative excipient composition is sufficiently similar to that of the comparator product, the physicochemical properties are comparable to ensure a similar microstructure, and the *in vitro* dissolution and release are similar. The WHO and INVIMA, Colombia’s guidelines, do not define requirements for emulsions/creams.

The physicochemical properties to compare the products may include those as for topical gels and ointments but with the addition of IVPT.

The possibility for a biowaiver to be accepted by the survey participants for topical dosage forms is summarised in Table 1.

**TABLE 1:** Comparison of biowaiver acceptance for topical dosage forms among IPRP BEWGG participants.

	AR	AU	BR	CA	CO	EU	IL	JP	MX	NZ	KR	SA	SG	ZA	CH	TW	UK	US	WHO
Are biowaivers acceptable?	Y	Y^a^	Y^b^	Y	Y	Y	Y^b^	N^c^	Y	Y^a^	Y	Y^d^	Y	Y^e^	Y^a^	Y	Y	Y^a^ ^,^ ^d^	Y
Topical solutions with the same or similar excipient composition	Y	Y^a^	Y	Y	Y	Y	Y	N	Y	Y^a^	Y	Y	Y	Y	Y^a^	Y	Y	Y	Y
Topical suspensions with the same or similar excipients	Y	Y^a^	Y	C	Y	Y	Y^b^	N	Y	Y^a^	Y	Y^d^	Y	Y	Y^a^	Y	Y	C^d^	Y^a^
Topical gels in solution with the same or similar excipients	Y	Y^a^	Y	Y	Y	Y	Y^b^	N	Y	Y^a^	Y	Y^d^	Y	Y	Y^a^	Y	Y	C^d^	Y
Topical gels in solution with systemic action	Y	Y^a^	Y	Y	C	Y	Y	N	Y	Y^a^	C	Y^d^	Y	C	Y^a^	Y	Y	C^d^	Y
Topical ointments with the same or similar excipients	Y	Y^a^	Y	Y	C^a^	Y	Y^b^	N	Y	Y^a^	Y	Y^d^	Y	Y	Y^a^	Y	Y	C	C^a^
Topical emulsions/creams with the same or similar excipients	Y	C^a^	Y	Y^a^	C^a^	Y	Y^b^	N	C	C^a^	Y	Y^d^	Y	Y	Y^a^	Y	Y	Y^d^	C^a^

AR: INVIMA, argentina; AU: TGA, australia; BR: ANVISA, brazil; CA: health canada, Canada; CH: swissmedic, Switzerland; CO: INVIMA, colombia; EU: EC, europe; IL: MOH, israel; JP: PMDA, japan; KR: MFDS, republic of korea; MX: COFEPRIS, mexico; NZ: medsafe, New Zealand; SA: SFDA, saudi arabia; SG: HSA, singapore; TW: TFDA, chinese taipei; UK: MHRA, united kingdom; US: FDA, united states; WHO: The World Health Organization (Observer) and ZA: SAHPRA, south africa.

Y: Yes. N: No. C: Case-by case.

^a^ Not defined in guidelines but may be acceptable based on sound scientific justification. Consultation with relevant agencies is recommended.

^b^ When defined in the product specific bioequivalence guidance.

^c^ Except corticosteroids for dermatological use.

^d^ Waivers acceptable based on alternatives approaches accepted by EMA, FDA, and WHO.

^e^ Biowaiver is acceptable in limited products (bactericides, disinfectants, and antiseptics) where the active site is the surface of the skin and the dose is not absorbed by the stratum corneum.

### Otic and ophthalmic products

#### Otic and ophthalmic solutions

All agencies, except PMDA, Japan, accept biowaivers for otic and ophthalmic solutions if the test and comparator products demonstrate similar Q1 and Q2. PMDA, Japan accepts waivers for ophthalmic solutions [46] and takes a case-by-case approach for otic solutions that are not described in their guidelines. In Australia [13], Canada [32], the EU [25], Japan [39], New Zealand [14], Republic of Korea [31, 32], Saudi Arabia [20], Singapore, South Africa [18], the UK, and the USA [21], supportive or comparative physicochemical data must be provided even when similar Q1 and Q2s are demonstrated (e.g., drop size, pH, osmolality, and viscosity). For the remaining participants, Q3 comparison is not necessary.

In the case where excipients vary qualitatively (e.g., preservative, buffer, substance to adjust tonicity, or thickening agent) but are similar quantitatively, a waiver for otic and ophthalmic solutions is still acceptable in Argentina, Canada, the EU, Mexico, New Zealand, Saudi Arabia, Singapore, South Africa, the Republic of Korea, Switzerland, and the USA if similar physicochemical properties can be shown (e.g., viscosity, surface tension, density, drop size, pH, buffer capacity, and osmolality) and it can be justified that safety, efficacy, and bioavailability are not affected. In contrast, INVIMA, Colombia; MOH, Israel; TFDA, Chinese Taipei; MHRA, UK; and the WHO accept such waivers, even if physicochemical properties are not similar, if it is justified that the safety, efficacy, or bioavailability is not affected. Australia would accept small quantitative differences in the same excipients if this does not affect absorption and physicochemical properties but would not accept qualitative changes in the excipients (in particular, changing benzalkonium chloride for another preservative). Similarly, a change in benzalkonium chloride for another preservative would not be acceptable in Canada, given that benzalkonium chloride has permeation-enhancing properties. ANVISA, Brazil would not accept a biowaiver for ophthalmic or otic aqueous solutions if any excipient is changed qualitatively, but quantitative changes are acceptable [9]. PMDA, Japan accepts a waiver for ophthalmic products only if the target tissue of active ingredient is the ocular surface and if the relevant animal model tests and/or *in vitro* tests meet the equivalence criteria.

#### Otic and ophthalmic suspensions

Waivers for otic or ophthalmic suspensions are not accepted in Brazil or Israel. In Japan, as with ophthalmic solutions, the waiver for ophthalmic suspensions is acceptable if the target tissue of the active ingredient is only the ocular surface and if relevant animal model tests and/or *in vitro* tests meet the equivalence criteria.

The WHO guideline “Multisource (generic) pharmaceutical products: guidelines on registration requirements to establish interchangeability” [16], which has also been adopted by ANMAT, Argentina and INVIMA, Colombia, recommends that otic and ophthalmic suspensions with the same qualitative and quantitative composition in excipients might be granted a waiver if the particles in suspension are shown to have the same crystallographic structure and similar particle size distribution, as well as comparability in any other appropriate *in vitro* test, e.g., dissolution. In Australia, Canada, the EU, New Zealand, Singapore, South Africa, Switzerland, the Republic of Korea, Chinese Taipei, and the UK, there is no specific guidance, but the participants will consider an application based on the test and comparator products being Q1 and Q2 and the physicochemical equivalence of justified parameters (e.g., in addition to the data required for solutions, the particle size distribution (PSD) of the drug in suspension, dissolution, etc.). The FDA, United States and SFDA, Saudi Arabia accept waivers for otic and ophthalmic suspensions on a case-by-case basis according to their product specific guidance [20, 21]. For example, ophthalmic suspensions of loteprednol [47] and fluorometholone [48] can be waived according to the corresponding product-specific BE guidance.

In case of qualitative and quantitative differences in excipient compositions, the same principles as described above for solutions apply (e.g., only quantitatively in Australia and Brazil). Some excipients that are justified not to affect the efficacy/activity of the drug or its safety profile can be changed qualitatively (e.g., preservative, buffer, substance to adjust tonicity, or thickening agent) in some participants as long it is demonstrated that those physicochemical properties (e.g., viscosity, surface tension, or pH) are not altered and equivalence is shown between test and comparator product to ensure that local bioavailability is not affected.

#### Otic and ophthalmic emulsions

Waivers of ophthalmic and otic emulsions for local action have not been described in regulatory guidelines, except for in the FDA, United States and SFDA, Saudi Arabia, product-specific guidance [20, 21] (e.g., cyclosporine [49] and difluprednate [50]). In these product-specific guidelines, a waiver (i.e., demonstration of equivalence based on *in vitro* data) may be possible if certain conditions are met (Q1, Q2, and Q3). This approach might be acceptable for all IPRP participants, except Japan and Brazil. If the generic is not Q1/Q2/Q3 to the comparator product, TE studies are generally required; however, a waiver of TE studies may be considered on a case-by-case basis if supported by compelling justification of relevance of the comparative product characteristics to the safety and efficacy of the formulation. For example, Health Canada, Canada has accepted this approach for cyclosporine.

The physicochemical parameters to be compared may include globule size distribution, viscosity profile as a function of applied shear, pH, zeta potential, osmolality and surface tension, as well as information on the drug distribution in different phases within the formulation.

#### Otic and ophthalmic ointments

The requirements are similar to those for topical ointments.

The possibility for a biowaiver to be accepted by the survey participants for otic and ophthalmic dosage forms is summarised in Table 2.

**TABLE 2:** Comparison of biowaiver acceptance for certain otic and ophthalmic dosage forms among IPRP BEWGG participants.

	AR	AU	BR	CA	CO	EU	IL	JP	MX	NZ	KR	SA	SG	ZA	CH	TW	UK	US	WHO
Ophthalmic solutions with the same excipients in the same amounts	Y	Y^a^	Y	Y^a^	Y	Y^a^	Y	Y^a^	Y	Y^a^	Y^a^	Y^a^	Y^a^ ^,^ ^b^	Y^a^	Y^a^	Y	Y^a^	Y^a^	Y
Otic solutions with the same excipients in the same amounts	Y	Y^a^	Y	Y^a^	Y	Y^a^	Y	C^b^	Y	Y^a^	Y^a^	Y^a^	Y^a^ ^,^ ^b^	Y^a^	Y^a^	Y	Y^a^	Y^a^	Y
Ophthalmic solutions with the same excipients in similar amounts	Y^c^	Y^a^	Y	Y^a^ ^,^ ^c^	Y^c^	Y^a^	Y	C^a^ ^,^ ^d^	Y^a^	Y^a^	Y^a^ ^,^ ^c^	Y^a^	Y^a^ ^,^ ^b^	Y^a^	Y^a^	Y^a^ ^,^ ^b^ ^,^ ^c^	Y^a^ ^,^ ^c^	Y^a^ ^,^ ^e^	Y^c^
Otic solutions with the same excipients in similar amounts	Y^c^	Y^a^	Y	Y^a^ ^,^ ^c^	Y^c^	Y^a^	Y	C^b^	Y^a^	Y^a^	Y^a^ ^,^ ^c^	Y^a^	Y^a^ ^,^ ^b^	Y^a^	Y^a^	Y^a^ ^,^ ^b^ ^,^ ^c^	Y^a^ ^,^ ^c^	Y^a^	Y^c^
Ophthalmic solutions with qualitative differences in some excipients (e.g., preservative, buffer, substance to adjust tonicity, or thickening agent)	Y^c^	N	N	Y^a^ ^,^ ^c^	Y^c^	Y^a^ ^,^ ^c^	Y^c^	C^a^ ^,^ ^d^	Y^a^ ^,^ ^c^	Y^a^ ^,^ ^c^	Y^a^ ^,^ ^c^	Y^a^ ^,^ ^c^	Y^a^ ^,^ ^b^ ^,^ ^c^	Y^a^ ^,^ ^c^	Y^a^ ^,^ ^c^	Y^a^ ^,^ ^b^ ^,^ ^c^	Y^a^ ^,^ ^c^	C	Y^c^
Otic solutions with qualitative differences in some excipients (e.g., preservative, buffer, substance to adjust tonicity, or thickening agent)	Y^c^	N	N	Y^a^ ^,^ ^c^	Y^c^	Y^a^ ^,^ ^c^	Y^c^	C^b^	Y^a^ ^,^ ^c^	Y^a^ ^,^ ^c^	Y^a^ ^,^ ^c^	Y^a^ ^,^ ^c^	Y^a^ ^,^ ^b^ ^,^ ^c^	Y^a^ ^,^ ^c^	Y^a^ ^,^ ^c^	Y^a^ ^,^ ^b^ ^,^ ^c^	Y^a^ ^,^ ^c^	C	Y^c^
Ophthalmic suspensions with the same excipients in the same amounts	Y^a^	Y^a^ ^,^ ^b^	N	Y^a^ ^,^ ^b^	Y^a^	Y^a^ ^,^ ^b^	N	C^a^ ^,^ ^d^	C	Y^a^ ^,^ ^b^	Y^a^	Y^a^ ^,^ ^e^	Y^a^ ^,^ ^b^	Y^a^ ^,^ ^b^	Y^a^ ^,^ ^b^	Y^a^ ^,^ ^b^	Y^a^ ^,^ ^b^	Y^a^ ^,^ ^e^	Y^a^
Otic suspensions with the same excipients in the same amounts	Y^a^	Y^a^ ^,^ ^b^	N	Y^a^ ^,^ ^b^	Y^a^	Y^a^ ^,^ ^b^	N	C^b^	C	Y^a^ ^,^ ^b^	Y^a^	Y^a^ ^,^ ^e^	Y^a^ ^,^ ^b^	Y^a^ ^,^ ^b^	Y^a^ ^,^ ^b^	Y^a^ ^,^ ^b^	Y^a^ ^,^ ^b^	Y^a^ ^,^ ^e^	Y^a^
Ophthalmic suspensions with the same excipients in similar amounts	Y^a^	Y^a^ ^,^ ^b^	N	Y^a^ ^,^ ^b^ ^,^ ^c^	Y^a^ ^,^ ^b^ ^,^ ^c^	Y^a^ ^,^ ^b^	N	C^a^ ^,^ ^d^	C	Y^a^ ^,^ ^b^	Y^a^ ^,^ ^c^	Y^a^ ^,^ ^b^ ^,^ ^e^	Y^a^ ^,^ ^b^	Y^a^ ^,^ ^b^	Y^a^ ^,^ ^b^	Y^a^ ^,^ ^b^ ^,^ ^c^	Y^a^ ^,^ ^b^ ^,^ ^c^	Y^a^ ^,^ ^e^	Y^a^ ^,^ ^b^ ^,^ ^c^
Otic suspensions with the same excipients in similar amounts	Y^a^	Y^a^ ^,^ ^b^	N	Y^a^ ^,^ ^b^ ^,^ ^c^	Y^a^ ^,^ ^b^ ^,^ ^c^	Y^a^ ^,^ ^b^	N	C^b^	C	Y^a^ ^,^ ^b^	Y^a^ ^,^ ^c^	Y^a^ ^,^ ^b^ ^,^ ^e^	Y^a^ ^,^ ^b^	Y^a^ ^,^ ^b^	Y^a^ ^,^ ^b^	Y^a^ ^,^ ^b^ ^,^ ^c^	Y^a^ ^,^ ^b^ ^,^ ^c^	Y^a^ ^,^ ^e^	Y^a^ ^,^ ^b^ ^,^ ^c^
Ophthalmic suspensions with qualitative differences in some excipients (e.g., preservative, buffer, substance to adjust tonicity, or thickening agent)	Y^a^	N	N	Y^a^ ^,^ ^b^ ^,^ ^c^	Y^a^ ^,^ ^b^ ^,^ ^c^	Y^a^ ^,^ ^b^ ^,^ ^c^	N	C^a^ ^,^ ^d^	C	Y^a^ ^,^ ^b^	Y^a^ ^,^ ^c^	C	Y^a^ ^,^ ^b^ ^,^ ^c^	Y^a^ ^,^ ^b^	Y^a^ ^,^ ^b^ ^,^ ^c^	Y^a^ ^,^ ^b^ ^,^ ^c^	Y^a^ ^,^ ^b^ ^,^ ^c^	C	Y^a^ ^,^ ^b^ ^,^ ^c^
Otic suspensions with qualitative differences in some excipients (e.g., preservative, buffer, substance to adjust tonicity, or thickening agent)	Y^a^	N	N	Y^a^ ^,^ ^b^ ^,^ ^c^	Y^a^ ^,^ ^b^ ^,^ ^c^	Y^a^ ^,^ ^b^ ^,^ ^c^	N	C^b^	C	Y^a^ ^,^ ^b^	Y^a^ ^,^ ^c^	C	Y^a^ ^,^ ^b^ ^,^ ^c^	Y^a^ ^,^ ^b^	Y^a^ ^,^ ^b^ ^,^ ^c^	Y^a^ ^,^ ^b^ ^,^ ^c^	Y^a^ ^,^ ^b^ ^,^ ^c^	C	Y^a^ ^,^ ^b^ ^,^ ^c^

Y: yes; N: no; C: case-by-case.

^a^ Additional *in vitro* comparisons are required.

^b^ Not defined in the guidelines.

^c^ Additional data to justify that the difference does not affect the safety and/or efficacy of the drug.

^d^ if defined in product specific guidance.

^e^ Biowaiver is acceptable if the target tissue of the active ingredient is the ocular and if relevant animal model tests and/or *in vitro* tests meet the equivalence criteria.

### Rectal and vaginal products

#### Enemas in solution

The possibility of biowaivers for enemas in solution is not described in the guidelines from ANMAT, Argentina; TGA, Australia; Health Canada, Canada; INVIMA, Colombia; PMDA, Japan; Medsafe, New Zealand; MFDS, Republic of Korea; Swissmedic, Switzerland; or the WHO. Notwithstanding the absence of guidance, the WHO would likely accept a biowaiver since an *in vivo* PK study is required only for non-solutions. Health Canada, Canada would also accept a biowaiver based on the principles described for aqueous solutions [33], i.e., Q1/Q2, physicochemical properties, and device attribute (if applicable) similarities. In the USA, product specific guidelines have been developed for some of these products, e.g., mesalamine [21, 51]. In the EMA guidelines on the investigation of bioequivalence [26], the Saudi Arabia Guidelines for Bioequivalence [52], and the ASEAN Guidelines for the Conduct of Bioequivalence Studies [27], which HSA, Singapore follows, it is stated “A waiver of the need to provide equivalence data may be acceptable in the case of solutions, e.g., eye drops, nasal sprays, or cutaneous solutions, if the test product is of the same type of solution (aqueous or oily) and contains the same concentration of the same active substance as the medicinal product currently approved”. In the EMA and TFDA, Chinese Taipei guidelines on equivalence studies for the demonstration of therapeutic equivalence for locally applied, locally acting products in the gastrointestinal tract [53, 54] it is also stated “If the test product is a solution at the time of administration and contains an active substance in the same concentration as an approved reference solution, studies supporting equivalent efficacy and safety may be waived.”

A waiver for enemas in solution would be considered by ANMAT, Argentina; TGA, Australia; ANVISA, Brazil; INVIMA, Colombia; EC, Europe; PMDA, Japan; COFEPRIS, Mexico; Medsafe, New Zealand; MFDS, Republic of Korea; SFDA, Saudi Arabia; HSA, Singapore; SAHPRA, South Africa; Swissmedic, Switzerland; TFDA, Chinese Taipei; MHRA, UK; and WHO if the excipients are Q1 and Q2. MOH, Israel does not accept waivers for enemas in solution even if the test and comparator products have the same components in the same amount (Q1/Q2).

The situation of a waiver for enemas in solution where the composition in excipients is not qualitatively or quantitatively the same/similar as in the comparator product is not described in the guidelines of ANMAT, Argentina; TGA, Australia; Health Canada, Canada; INVIMA, Colombia; PMDA, Japan; Medsafe, New Zealand; MFDS, Republic of Korea; HSA, Singapore; Swissmedic, Switzerland; or the WHO. In contrast, in the EMA and TFDA, Chinese Taipei guidelines [53, 54] it is stated that “particular consideration should be given to the amount and type of excipients that may affect local tolerance, local residence time (e.g., surface tension, viscosity, etc.), *in vivo* solubility (e.g., co-solvents), or *in vivo* stability of the active substance. Minor differences in the excipient composition may be acceptable if the relevant pharmaceutical properties of the test product and comparator product are identical or essentially similar. Any qualitative or quantitative differences in excipients must be satisfactorily justified in relation to their influence on therapeutic equivalence. The method and means of administration should also be the same as the medicinal product currently approved, unless otherwise justified”. The SFDA, Saudi Arabia [52] and ASEAN guidelines [27] state “Minor differences in the excipient composition may be acceptable if the relevant pharmaceutical properties of the test product and reference product are identical or essentially similar in case of solutions products, e.g., eye drops, nasal sprays, or cutaneous solutions.” The WHO does not have a specification in their guideline either, but a waiver would be possible in other administration routes if the differences in excipients are considered to not affect bioavailability. Again, the principles of the acceptance criteria described in the Health Canada guidance [33] would apply to a waiver with respect to Q1/Q2, physicochemical properties, and device attribute (if applicable) similarities such that a waiver would not be accepted if the requirements were not met. The Health Canada guidance also indicates that differences beyond the criteria could be scientifically justified based on the impact on safety, efficacy, and absorption. The same acceptance criteria apply in Australia, Colombia, Japan, Mexico, New Zealand, the Republic of Korea, South Africa, Switzerland, and the UK on a case-by-case basis. ANMAT, Argentina and ANVISA, Brazil accept such a waiver as well, without further conditions. MOH, Israel does not accept a waiver as explained above for enemas in solution and the USA does not accept waivers if the excipient compositions changes outside the limits of Q1 and Q2 [36].

#### Enemas in suspension

Similar to the situation for biowaivers for enemas in solution, the possibility of biowaivers for enemas in suspension are not described in the guidelines from Argentina, Australia, Brazil, Canada, Colombia, Israel, Japan, Mexico, New Zealand, the Republic of Korea, Singapore, South Africa, Switzerland, the UK, or the WHO.

In Argentina, Australia, Canada, Colombia, Japan, Mexico, New Zealand, the Republic of Korea, Saudi Arabia, Singapore, South Africa, Switzerland, and the UK and from the WHO, a waiver for enemas in suspension would be possible on a case-by-case basis if the drug product was locally acting, without systemic exposure, and was similar with respect to Q1, Q2, and Q3. In the USA, the recommendations are defined in some general [21, 22, 36] and product-specific guidance.

In the EMA and TFDA, Chinese Taipei guidelines on equivalence studies for the demonstration of therapeutic equivalence for locally applied and acting products in the gastrointestinal tract [53, 54] it is stated “If the test product is not a solution (e.g., solid dosage form), demonstration of equivalent drug release and availability at the sites of action can be considered as surrogate of therapeutic equivalence. In those cases where systemic bioavailability is observed, a PK BE study is required in order to address systemic safety, unless otherwise justified. In such cases plasma levels could also be used as a surrogate of equivalence in efficacy for products acting locally in the rectum and the colon (e.g., enemas) if the drug is absorbed from the sites of action. Then, plasma levels reflect the drug release and availability close to the sites of action. Comparison of drug levels in faeces may be necessary.” Therefore, a biowaiver is not acceptable in principle. As soon as systemic exposure is measurable, a PK study would be required for safety and efficacy. Similarly, in Brazil, Canada, Colombia, Israel, Japan, Saudi Arabia, South Africa, Switzerland, the UK, the US, and for the WHO, such a waiver is not accepted.

If a PK study is not feasible because plasma levels are not measurable, a therapeutic equivalence study with a clinical endpoint is requested in the EU. However, the company could always justify a deviation from the guideline if the clinical/PD endpoints are insensitive to detect differences and the *in vitro* properties employed to support a biowaiver are shown to be more discriminative than the clinical/PD endpoints. A comparison of the crystallography and particle size distribution, *in vitro* dissolution, viscosity, surface tension, etc. could be considered.

If the composition in excipients is not qualitatively or quantitatively the same as the comparator product, a waiver may still be considered by COFEPRIS, Mexico if the differences in excipients are considered not to affect bioavailability. A waiver would not be acceptable for the remaining participants; however, if the differences in excipients are considered not to affect the bioavailability, an *in vivo* PK study is not feasible and the PD or clinical endpoints are known to be insensitive due to a flat dose-response curve, an *in vitro* comparison would be considered if it is justified that the excipient differences do not affect local bioavailability (e.g., differences are only in non-functional excipients like preservatives that do not affect physicochemical properties). At this point in time, none of the participating agencies have specified a criterion to define the difference between excipients to grant a waiver. The FDA, United States may allow for Drug Efficacy Safety Implementation – a waiver for certain enemas such as Sodium Polystyrene Sulfonate [21]; however, the criteria for consideration is based on different criteria, as outlined in FDA regulations [21].

Most participants have not defined what *in vitro* tests are required to compare test and comparator products but should include the *in vitro* tests used for the characterisation of solutions and suspensions. TGA, Australia; Health Canada, Canada; TFDA, Chinese Taipei; INVIMA, Colombia; EC, Europe; COFEPRIS, Mexico; Medsafe, New Zealand; SFDA, Saudi Arabia; HSA, Singapore; SAHPRA, South Africa; Swissmedic, Switzerland; MHRA, UK; and the WHO would request data to confirm Q1, Q2, and similar physicochemical properties, which might include viscosity and smear tests, pH, surface tension, osmolarity, drug substance morphology, and particle size distribution as well as dissolution profiles. As soon as excipients differ slightly, it would be necessary to verify surface tension, viscosity, pH, osmolality, etc. for solutions and, additionally, crystallography, dissolution profile, and PSD would be requested for suspensions.

The list of dosage forms included in this review is not exhaustive. For example, the requirements to demonstrate equivalence for rectal or intra-anal ointments are not described. However, similar requirements to those applied for topical ointments might be applied. A waiver is possible for nitroglycerine intra-anal ointment according to the corresponding FDA product-specific guidance [55].

#### Suppositories

Many participants like ANMAT, Argentina; TGA, Australia; ANVISA, Brazil; Health Canada, Canada; INVIMA, Colombia; MOH, Israel; PMDA, Japan; COFEPRIS, Mexico; Medsafe, New Zealand; MFDS, Republic of Korea; HSA, Singapore; SAHPRA, South Africa; Swissmedic, Switzerland; and the WHO do not describe biowaivers for these dosage forms in their guidelines. In the EU, UK, Saudi Arabia, and ASEAN, suppositories for systemic action are considered in the guidelines on the investigation of bioequivalence [26, 27] where it is stated that for non-oral immediate release dosage forms with systemic action, e.g., rectal formulations, in general, bioequivalence studies are required. Those suppositories with local action are considered in the EMA guideline on equivalence studies for the demonstration of therapeutic equivalence for locally applied, locally acting products in the gastrointestinal tract [53] and, as solid dosage forms, a waiver as described above for suspensions is not acceptable. In the USA, the recommendations are described in some general guidelines [21, 22, 36] and product-specific guidance, where waivers for suppositories are not accepted. The WHO [16] and INVIMA, Colombia [17] require bioequivalence studies for systemically acting products and therapeutic equivalence trials for locally acting products. All participants state that they would not accept waivers for suppositories when they work after systemic absorption. Therefore, the biowaivers, if any, would be limited to locally acting products with no measurable systemic exposure.

ANMAT, Argentina; TGA, Australia; ANVISA, Brazil; TFDA, Chinese Taipei; MOH, Israel; PMDA, Japan; COFEPRIS, Mexico; Medsafe, New Zealand; MFDS, Republic of Korea; SFDA, Saudi Arabia; HSA, Singapore; SAHPRA, South Africa; and Swissmedic, Switzerland do not have any experience so far in assessing biowaivers for locally acting suppositories. If waivers were to be accepted for locally acting suppositories with no measurable systemic exposure, TGA, Australia; Health Canada, Canada; TFDA, Chinese Taipei; EC, Europe; MOH, Israel; Medsafe, New Zealand; SFDA, Saudi Arabia; SAHPRA, South Africa; Swissmedic, Switzerland; and the MHRA, UK would require Q1, Q2, Q3, and an IVRT.

In Argentina, Brazil, Mexico, and South Africa, a waiver could be possible even if the excipients are different, but the different excipients should have the same function proof should be provided that the differences in excipients do not affect product performance.

The physicochemical properties such as melting point, speed, and breaking strength should be compared. Additionally, the same crystallography and PSD would be required in case of suspensions. ANVISA, Brazil would apply pharmacopoeial requirements and COFEPRIS, Mexico would act on a case-by-case basis depending on the type of product, as defined by “Consejo de Salubridad General”, which is a body of the Ministry of Health that advises COFEPRIS on these technical requirements.

#### Vaginal solid dosage forms

The possibility of biowaivers for vaginal solid dosage forms (e.g., ovules, capsules, or tablets) is not considered in any guideline. In the USA, the applicable guidelines would be those of topical products if locally acting [21, 22, 36], but no product-specific guidance has described the possibility of a waiver for these dosage forms. Therefore, *in vivo* BE with PK, PD, and/or clinical endpoints studies are in principle required. The only participants that could consider waivers on a case-by-case basis for vaginal solid dosage forms are ANMAT, Argentina; TGA, Australia; ANVISA, Brazil; Health Canada, Canada; INVIMA, Colombia; EC, Europe; PMDA, Japan; COFEPRIS, Mexico; Medsafe, New Zealand; SFDA, Saudi Arabia; HSA, Singapore; SAHPRA, South Africa; Swissmedic, Switzerland; TFDA, Chinese Taipei; MHRA, UK; and the WHO; however, only Health Canada, Canada has received a biowaiver application, which is pending review. In the WHO Prequalification Programme, the waiver for the vaginal route of administration has been proposed for misoprostol tablets if excipients are qualitatively the same, quantitatively similar, and the *in vitro* properties are equivalent as to obtain a BCS-based biowaiver for the oral route [56]. Therefore, a case-by-case approach is applied. In Israel, the Republic of Korea, and the USA, a waiver is not possible.

For members that could accept waivers on a case-by-case basis, different criteria may be applied. If the drug is systemically acting or it is locally acting but plasma levels are measurable, it is more probable that an *in vivo* PK study would be required. On the contrary, if the product is locally acting without measurable systemic levels and the dose-response curve is flat, the acceptance of a waiver is more probable.

Similarly, if the excipients are Q1 and Q2, the biowaiver is more likely than if excipients are not Q1 and Q2. Australia would require appropriate physicochemical data in case of small Q2 differences. In Brazil and Mexico, any qualitatively and quantitatively changed excipients should be justified by the sponsor.

Similarly, a waiver for vaginal solid dosage forms where the drug is in suspension would be more unlikely than where it is in solution.

Comparative *in vitro* tests between the test and comparator products would be required by TGA, Australia; EC, Europe; COFEPRIS, Mexico; Medsafe, New Zealand; SFDA, Saudi Arabia; HSA, Singapore; Swissmedic, Switzerland; MHRA, UK; and the WHO PQP; however, given that guidelines do not exist for the participants, the concerned agency should be consulted to determine the type of parameters that should be conducted for Q3 testing. For example, TGA, Australia; EC, Europe; HSA, Singapore; and MHRA, UK would require comparative physicochemical testing for melting point, disintegration, dissolution, polymorphic form, and particle size (if in suspension). ANVISA, Brazil and ANMAT, Argentina would ask the sponsor to show pharmacopoeial requirements to compare the test and comparator product. Health Canada, Canada would require demonstration of Q1 and Q2 as well as comparative dissolution for an ovule, tablet, or capsule that was inserted manually. If a device is used to administer the dosage form, demonstration that the device is sufficiently similar with respect to the design, physical dimensions, and material of construction to the comparator product, such that there is no change to the labelling of in-use instructions, would be required. In addition, demonstration of compatibility/biological activity and potential extractable and leachables may also be required.

The possibility for a biowaiver to be accepted by the survey participants for rectal and vaginal dosage forms is summarised in Table 3.

**TABLE 3:** Comparison of Biowaiver Acceptance for rectal (enemas and suppositories) and vaginal solid dosage forms Among IPRP BEWGG Participants.

	AR	AU	BR	CA	CO	EU	IL	JP	MX	NZ	KR	SA	SG	ZA	CH	TW	UK	US	WHO
Enemas in solution with the same excipients in the same amounts	Y^a^	Y^a^	Y	Y^a^	Y^a^	Y	N	C^a^	Y	Y^a^	Y^a^	Y	Y	Y	Y^a^	Y	Y	C^b^	Y^a^
Enemas in solution with differences in excipients	Y^a^	C^a^	Y	N^a^	Y^a^	Y	N	C^a^	C	Y^a^	C^a^	Y	Y^a^	Y	C^a^	Y	Y	N	Y^a^
Enemas in suspension Q1 and Q2 with systemic exposure	C^a^	C^a^	N^a^	N^a^	N^a^	N	N^a^	N^a^	C^a^	C^a^	C^a^	N^a^	N	N^a^	N^a^	N	N^a^	N^b^	N^a^
Enemas in suspension Q1 and Q2 without systemic exposure	C^a^	C^a^	N^a^	C^a^	C^a^	C	N^a^	C^a^	C^a^	C^a^	C^a^	C^a^	C^a^	C^a^	C^a^	C	C^a^	C^b^	C^a^
Enemas in suspension without Q1 and Q2	C^a^	N^a^	N^a^	N^a^	N^a^	N	N^a^	C^a^	C^a^	N^a^	C^a^	N^a^	N	N^a^	N^a^	N	N^a^	N	N^a^
Suppositories with systemic action	N^a^	N^a^	N^a^	N^a^	N^a^	N	N^a^	N^a^	N^a^	N^a^	N	N	N	N^a^	N^a^	N^a^	N^a^	N	N^a^
Suppositories for local action	Y^a^	C^a^	C^a^	C^a^	N^a^	C	C^a^	C^a^	Y^a^	C^a^	N	C^a^	C^a^	C^a^	C^a^	C	N^a^	N	N^a^
Vaginal solid dosage forms for systemic action	C^a^	N^a^	C^a^	C^a^	C^a^	N^a^	N^a^	N^a^	N^a^	C^a^	N	N^a^	N^a^	C^a^	N^a^	N^a^	N^a^	N	C^a^
Vaginal solid dosage forms for local action	Y^a^	C^a^	C^a^	C^a^	C^a^	C^a^	N	C^a^	Y^a^	C^a^	N	C^a^	C^a^	C^a^	C^a^	C^a^	C^a^	N	C^a^

Y: yes; N: no; C: case-by-case.

^a^ Not defined in the guidelines.

^b^ if defined in product specific guidance.

### Statistical comparison of the physicochemical parameters (test vs. comparator)

Another important topic is the type of statistical analysis to be conducted to compare physicochemical properties. For some participants, a formal statistical analysis is not required for same parameters (e.g., FDA, United States) [22], whereas in others, the use of 90% confidence intervals of average BE are employed with or without log-transformation of the data for the ratio T/R or the difference T-R [53]. In the USA, a population BE (PBE) approach is employed for particle size distribution [47]. For example, for locally acting drug products such as topicals, establishing BE relies on the evaluation of comparative physicochemical properties, with some attributes (e.g., appearance, pH, or specific gravity) not requiring statistical analysis [22]; however, for characteristics like drug particle and particle size distribution, population bioequivalence (PBE) analysis is employed [46], as it accounts for both within- and between-batch variability, unlike average BE [53], which focuses on mean differences.

Finally, the number of batches to be tested in the comparison is usually not defined, except in case of product-specific guidelines. Typically, where indicated (e.g., in product-specific guidelines), data is requested for three lots of the test and comparator products. A larger sample size may be required in case of significant inter-batch variability or high complexity in the formulation (e.g., Health Canada, Canada). The number of replicates per batch is generally not defined, but a minimum of three replicates is expected. The number of replicates should be based on the intra-batch variability and the type of statistical analysis. For example, in the PBE analysis employed to compare the PSD of cyclosporine ophthalmic emulsions, no less than 10 datasets from three batches each of the test and reference standard are to be used [49].

## Discussion

This survey included the biowaiver recommendations and requirements for three categories of systemically and locally acting products based on site of application: 1) topical products; 2) otic and ophthalmic products; and 3) rectal and vaginal products. In general, the results show that some, if not most, of the members do not have guidance documents that clearly define the waiver recommendations or requirements from conducting *in vivo* studies for these dosage forms. Most members would grant biowaivers for topical products, otic products, and ophthalmic products, albeit subject to diverse criteria; however, as the dosage forms become more complex (e.g., from solutions to suspensions and emulsions), the eligibility for qualifying for a biowaiver becomes less likely or “case-by-case”. The complexity of a dosage form is dependent on whether the drug is in solution or a vehicle (e.g., suspension, cream, or gel), dependent on whether the drug acts locally or systemically, and whether excipients are responsible for drug release and/or absorption. Given the complexity of the dosage forms, case-by case assessment regarding the eligibility of the dosage form for a biowaiver is contingent on the inherent variability of specific criteria that are applied by varying regulatory authorities. A similar observation was previously reported for systemically acting oral and injectable dosage forms [6].

There was a high degree of convergence among the survey participants for generic topical, ophthalmic, and otic solutions with the same qualitative and quantitative composition as the comparator products; because the drug is in solution and already released, the same excipient composition in the solution ensures that the bioavailability and tolerability at the site of action will be the same between the generic and the comparator product, and drug absorption cannot be modified by the method of manufacture. In this context, convergence among all participants is only found in the case of ophthalmic aqueous solutions. All participants would consider granting a biowaiver for otic aqueous solutions or topical solutions with the same qualitative (Q1) and similar quantitative (Q2) composition as the comparator product, except PMDA, Japan. PMDA, Japan follows a case-by-case approach for otic products and only accepts biowaivers for a very limited range of topical solutions (bactericides, disinfectants, and antiseptics that exert their effect on the skin and in which the drug is not absorbed by the stratum corneum). Interestingly, more than half of the participants require the comparison of *in vitro* physicochemical properties (Q3) even when the excipient compositions of the generic and comparator product are identical. If there are qualitative differences and quantitative differences that are out of the acceptable range of similarity, participants are also likely to request additional data to justify the lack of effects on the efficacy and safety of the drug, particularly for ophthalmic and otic products. In spite of this, TGA, Australia and ANVISA, Brazil do not grant waivers when there are qualitative excipient differences for otic and ophthalmic products; PMDA, Japan and FDA, United States generally follow a case-by-case approach. In general, the larger the differences in excipient composition, the lower the probability for a successful biowaiver.

As the dosage forms increase in complexity away from aqueous solutions, the divergence in recommendations and requirements also increases. Topical gels not intended for systemic action and without expected systemic adverse reactions are considered slightly more complex than aqueous solutions. If the drug is in solution, the gel could be considered as a “very viscous” solution, therefore, a biowaiver may be possible in most of the participating jurisdictions that allow biowaivers for topical solutions. Consistent with its regulatory practice for topical solutions, Japan does not accept a biowaiver for topical gels. SFDA, Saudi Arabia and FDA, United States only grant biowaivers when there is existing product-specific guidance and MOH, Israel does not grant biowaivers for topical gels containing corticosteroids for dermatological use.

Topical, otic, and ophthalmic ointments are handled similarly to topical gels as the drug is also in solution. Hence, the acceptability of biowaivers is the same as for topical gels except for with INVIMA, Colombia and the WHO, which follow a case-by-case approach. The assessment of qualitative and quantitative differences in excipient composition is almost identical between topical gels and topical solutions, while being somewhat similar to that for ointments.

Suspensions are also a more complicated dosage form when compared to solutions. Biowaivers for topical suspensions tend to be accepted in a larger number of countries when compared to oral and injectable suspensions. The same participants that accept biowaivers for topical solutions also accept biowaivers for topical suspensions, with the exception of Health Canada, Canada and FDA, United States, who follow a case-by-case approach. With the exception of ANVISA, Brazil and MOH, Israel, almost half of the participants would accept a biowaiver for ophthalmic and otic suspensions if the generic has the same qualitative composition and same or similar quantitative composition to the comparator product. PMDA, Japan and COFEPRIS, Mexico follow a case-by-case approach and SFDA, Saudi Arabia and FDA, United States generally allow biowaivers only if defined in product-specific guidelines. When there are qualitative differences, TGA, Australia would not accept a biowaiver and SFDA, Saudi Arabia and the FDA, United States would consider a biowaiver on a case-by-case basis. There is a large discrepancy among the participants regarding the *in vitro* tests that are required to demonstrate *in vitro* comparability.

Emulsions are considered the most complex formulations administered via the topical, ophthalmic, and otic routes. Many participants do not have specific guidelines for emulsions and are more likely to critically assess the differences in excipient composition between the generic and the comparator product and follow a case-by-case approach towards granting biowaivers.

The waiver for rectal and vaginal solid dosage forms is not described in guidelines for most participants. The is a greater likelihood of obtaining a biowaiver for enemas where the drug is in solution if Q1/Q2 similarity is demonstrated between the generic and comparator product. All participants would grant biowaivers in this case except MOH, Israel, while PMDA, Japan and FDA, United Stated would consider granting a biowaiver on a case-by-case basis. When there are excipient differences, Health Canada, Canada would not accept a biowaiver for an enema in solution, while TGA, Australia; COFEPRIS, Mexico; MFDS, Republic of Korea; and Swissmedic, Switzerland take a case-by-case approach. The other participants may accept justifications that the differences are irrelevant based on *in vitro* comparisons if the excipients are not functional. For all other rectal and vaginal dosage forms, biowaivers are either not granted or are assessed on a conservative case-by-case basis. Interestingly, in South Africa, oral suspensions with measurable levels could be waived but enemas in suspensions with systemic action cannot be waived. None of the members grant biowaivers for suppositories that are systemically acting, but eight of the 20 participants would follow a case-by-case approach for vaginal solid dosage forms with systemic action. Overall, a biowaiver is most likely to be granted for rectal and vaginal dosage forms in which the drug is locally acting and in solution and has the same qualitative and quantitative composition as the comparator product.

A notable scenario is the case of topical products containing corticosteroids, where a validated pharmacodynamic (PD) model exists (i.e., blanching or vasoconstriction assay). ANVISA, Brazil and MOH, Israel do not accept biowaivers for these products as they consider the PD data to be more reliable than *in vitro* data used to support a biowaiver. In the EMA draft guideline, this PD study was also requested for corticosteroids, in addition to the *in vitro* data for the waiver, but this is under review for the final guideline. In the EU, a stepwise approach is to be employed (i.e., step 1 based on *in vitro* data, step 2 based on kinetic data (PK, IVPT, or TS), and step 3 based on PD or clinical endpoints). When the conditions for a waiver are fulfilled, the *in vitro* data are considered more discriminative or sensitive than the PD data. The availability of a validated PD model offers an alternative to the clinical endpoints when the waiver is not possible in the first and second steps.

The results of the survey also demonstrated that there is a lack of convergence regarding the limits for qualitative and quantitative differences between the generic and the comparator product for topical, rectal, and vaginal dosage forms due to the absence of predefined criteria in most of the existing guidelines. At present, only Health Canada, Canada has specified a 10% limit for quantitative differences for aqueous solutions, whereas in the USA a 5% difference is usually accepted. While not finalized, the EU guidelines may provide acceptance criteria for qualitative and quantitative differences larger than those defined in the current draft if it is shown that the differences do not affect the local availability, the physicochemical properties, and/or other aspects of the therapeutic effect that depend on the vehicle itself; however, in principle, when the differences are larger than what is considered as similar, the waiver is not possible.

Similarly, there is divergence in the type of *in vitro* tests required to demonstrate *in vitro* comparability and the acceptance criteria for these tests is due to a general absence of guidance for topical, rectal, and vaginal products, except for the product-specific bioequivalence guidelines published by the FDA, United States, and SFDA. Saudi Arabia. Furthermore, topical products are very diverse within the categories identified by the traditional names of the dosage forms (e.g., creams, lotions, gels, and ointments).

Therefore, further scientific discussion is required to achieve further convergence and even harmonisation at the ICH level. Many of the BEWGG members are individually assessing the *in vitro* physicochemical parameters that are required to support the biowaivers for each dosage form. It would be much more productive if the respective agencies assembled to discuss the recommendations and requirements in this rapidly developing and complex field. The product specific guidance from SFDA, Saudi Arabia and FDA, United States would be an excellent launching point for a basis of scientific discussion. This review has provided an introduction to the principles applied for each of the dosage forms to facilitate the extension of biowaivers to other dosage forms.

## Conclusion

Guidelines for biowaivers have not been developed by a majority of IPRP participating regulators (e.g., 12/19 as of 2023) for most of the dosage forms such as cutaneous/topical products [creams, ointments], otic/ophthalmic suspensions, and enemas and vaginal suppositories. As many of the dosage forms are considered complex, fewer generics are developed compared to oral or injectable forms due to the lack of regulatory guidance in many jurisdictions and the higher risks of non-acceptance by regulators. The results of the survey and technical discussions of the IPRP BEWGG mark the first step towards regulatory convergence in the area of biowaivers for non-oral dosage forms and highlights the differences in biowaiver requirements among members of the IPRP BEWGG. For participants (e.g., ANVISA, Health Canada) who are willing to consider biowaivers on a case-by-case basis, there is a risk that the scientific justifications submitted by generic drug manufacturers may result in different outcomes in different countries or regions. The development of guidelines that describe when biowaivers are eligible (e.g., BCS-based waivers for non-absorbed enemas) or when *in vivo* bioequivalence studies are required to demonstrate equivalence (via PK, PD, or clinical endpoints) would facilitate the drug application process for multiple regulatory authorities. The challenge with developing harmonized guidance is that many of the smaller regulators have had limited experience with the aforementioned dosage forms (e.g., vaginal tablets or otic suspensions). There are instances where guidance is available from one regulatory authority (e.g., CHMP/QWP/708282/2018 [25]) and other participants may adapt the guidance to their own needs or interpretations (e.g., differences in the statistical approaches). On the contrary, when guidance documents have been issued by many different members (e.g., EC, Europe and FDA, United States), convergence can sometimes become more difficult. Nevertheless, it is undeniable that convergence in this area would be useful for pharmaceutical companies developing generic medicinal products for approval in all participating jurisdictions.

## Data Availability

The original contributions presented in the study are included in the article/supplementary material, further inquiries can be directed to the corresponding author.

## References

[1] International pharmaceutical regulators programme (2023) Available online at: http://www.iprp.global/home (Accessed March 19, 2024).

[2] van Oudtshoorn J García-Arieta A Santos GML Crane C Rodrigues C Simon C A survey of the regulatory requirements for BCS-based biowaivers for solid oral dosage forms by participating regulators and organisations of the international generic drug regulators programme. J Pharm Pharm Sci (2018) 21(1):27–37. 10.18433/J3X93K 29382433

[3] ICH Harmonised Guideline. Biopharmaceutics classification System-based biowaivers (M9) (2023). Available online at: https://database.ich.org/sites/default/files/M9_Guideline_Step4_2019_1116.pdf (Accessed March 19, 2024).

[4] Crane C Santos GML Fernandes EAF Simon C Tam A Triana DG The requirements for additional strength biowaivers for immediate release solid oral dosage forms in international pharmaceutical regulators programme participating regulators and organisations: differences and commonalities. J Pharm Pharm Sci (2019) 22(1):486–500. 10.18433/jpps30724 33760728

[5] Roost MS Potthast H Walther C García-Arieta A Abalos I Agostinho Freitas Fernandes E Requirements for additional strength biowaivers for modified release solid oral dosage forms in international pharmaceutical regulators programme participating regulators and organisations: differences and commonalities. J Pharm Pharm Sci (2021) 24:548–62. 10.18433/jpps32260 34706215

[6] Garcia Arieta A Simon C Tam A Mendes Lima Santos G Freitas Fernandes EA Rodríguez Martínez Z A survey of the regulatory requirements for the waiver of *in vivo* bioequivalence studies of generic products in certain dosage forms by participating regulators and organisations of the international pharmaceutical regulators programme. J Pharm Pharm Sci (2021) 24:113–26. 10.18433/jpps31491 33734975

[7] Ministry of Health, Labour and Welfare (MHLW), Japan. Guideline for bioequivalence studies of generic products for topical use (2003). Available online at: http://www.nihs.go.jp/drug/be-guide(e)/Topical_BE-E.pdf (Accessed on December 22, 2023).

[8] Ministry of Health, Labour and Welfare (MHLW), Japan. Guideline for bioequivalence studies of generic products for topical use Q&A (2006). Available online at: https://www.pmda.go.jp/files/000161027.pdf (Accessed December 22, 2023).

[9] Agência Nacional de Vigilância Sanitária (ANVISA) (BRAZIL). RDC n.749, of 5th September 2022. Brazil: Provides for Biowaiver (2022). Available online at: http://antigo.anvisa.gov.br/documents/10181/2695968/%281%29RDC_749_2022_.pdf/b0f1492b-f22d-456b-a61d-634cd5a8d2c7 (Accessed December 22, 2023).

[10] Administración Nacional de Medicamentos, Alimentos y Tecnología Médica (ANMAT). Disposición ANMAT N° 3185 de 1999. Recomendaciones técnicas para la realización de estudios de equivalencia entre medicamentos de riesgo sanitario significativo (2023). Available online at: https://www.argentina.gob.ar/normativa/nacional/disposici%C3%B3n-3185-1999-58457/actualizacion (Accessed December 22, 2023).

[11] Comisión Federal para la Protección contra Riesgos Sanitarios (COFEPRIS). Article 2, fraction XIV Bis, of the Reglamento de Insumos Para la Salud, (Health Products Regulation) (2023). Available online at: http://www.diputados.gob.mx/LeyesBiblio/regley/Reg_LGS_MIS.pdf (Accessed December 22, 2023).

[12] Health Sciences Authority (Singapore). Appendix 10 product interchangeability and biowaiver request for chemical generic applications, guidance on medicinal product registration in Singapore (April 2022) (2023). Available online at: https://www.hsa.gov.sg/docs/default-source/hprg-tpb/guidances/appendix-10_product-interchangeability-and-biowaiver-request-for-chemical-generic-drug-applications.pdf (Accessed on December 22, 2023).

[13] Therapeutic Goods Administration (Australia). Biopharmaceutic studies previously guidance 15: biopharmaceutic studies (version 1.2, December 2019) (2023). Available online at: https://www.tga.gov.au/sites/default/files/guidance-15-biopharmaceutic-studies.pdf (Accessed on December 24, 2023).

[14] Medsafe (New Zealand). Part 6: bioequivalence of medicines, guideline on the regulation of therapeutic products in New Zealand (2015). Available online at: http://www.medsafe.govt.nz/regulatory/Guideline/GRTPNZ/bioequivalence-of-medicines.pdf (Accessed on December 24, 2023).

[15] Ministry of Health (Israel). Guideline no 45, 46 guideline for submission applications for registration, variation and renewal to registration department, pharmaceutical division (REG 08_2012) (2023). Available online at: https://www.health.gov.il/hozer/Reg08_2012.pdf (Accessed December 22, 2023).

[16] Multisource (generic) pharmaceutical products: guidelines on registration requirements to establish interchangeability. Annex 6. In TRS 1003. Fifty-first report. (2017). Available online at: https://www.who.int/publications/m/item/annex-6-trs-1003 (Accessed December 24, 2023).

[17] Instituto Nacional de Vigilancia de Medicamentos y Alimentos (Colombia). Resolución 1124 de 2016. Por la cual se establece la Guía que contiene los criterios y requisitos para el estudio de Biodisponibilidad y Bioequivalencia de medicamentos, se define el listado de los que deben presentarlos y se establecen las condiciones de las Instituciones que los realicen (2016). Available online at: https://www.minsalud.gov.co/sites/rid/Lists/BibliotecaDigital/RIDE/DE/DIJ/resolucion-1124-de-2016.pdf (Accessed on December 24, 2023).

[18] Medicines Control Council (South Africa). Biostudies (june 2015) (2023). Available online at: https://www.sahpra.org.za/wp-content/uploads/2020/01/61de452d2.06_Biostudies_Jun15_v6.pdf (Accessed on December 24, 2023).

[19] Swissmedic. Guidance document: authorisation of human medicinal product with known active pharmaceutical substance HMV4 (2023). Available online at: https://www.swissmedic.ch/dam/swissmedic/en/dokumente/zulassung/zl_hmv_iv/zl101_00_007d_wlanleitungzulassungvonhumanarzneimittelnmitbekann.pdf.download.pdf/ZL101_00_007e_WL%20Guidance%20document%20Authorisation%20of%20human%20medcine%20with%20known%20active%20pharmaceutical%20ingredient.pdf (Accessed on December 24, 2023).

[20] Saudi Food and Drug Authority (SFDA). SFDA’s product specific bioequivalence guidance (version 1.0) (2023). Available online at: https://www.sfda.gov.sa/sites/default/files/2022-05/SFDAsProductSpecificBioequivalenceGuidanceV1.pdf (Accessed December 22, 2023.

[21] U.S. Department of Health and Human Services. Food and Drug Administration. Center for Drug Evaluation and Research (CDER). Product-specific guidances for generic drug development (2023). Available online at: https://www.fda.gov/drugs/guidances-drugs/product-specific-guidances-generic-drug-development (Accessed on December 22, 2023).

[22] U.S. Department of Health and Human Services. Food and Drug Administration. Center for drug evaluation and research (CDER) physicochemical and structural (Q3) characterization of topical drug products submitted in ANDAs. Draft guidance (2022). Available online at: https://www.fda.gov/media/162471/download (Accessed on December 22, 2023).

[23] U.S. Department of Health and Human Services. Food and Drug Administration. Center for drug evaluation and research (CDER). In: Vitro release test studies for topical drug products submitted in ANDAs. Draft guidance (2022). Available online at: https://www.fda.gov/media/162476/download (Accessed on December 22, 2023).

[24] U.S. Department of Health and Human Services. Food and Drug Administration. Center for Drug Evaluation and Research (CDER). *In Vitro* permeation test studies for topical drug products submitted in ANDAs guidance for industry. United States: Draft Guidance (2022). Available online at: https://www.fda.gov/media/162475/download (Accessed on December 22, 2023).

[25] Committee for Medicinal Products for Human Use (CHMP). Draft guideline on quality and equivalence of topical products (CHMP/QWP/708282/2018) (2018). Available online at: https://www.ema.europa.eu/en/documents/scientific-guideline/draft-guideline-quality-equivalence-topical-products_en.pdf (Accessed on December 22, 2023).

[26] Committee for Medicinal Products for Human Use (CHMP). Guideline on the investigation of bioequivalence (CPMP/EWP/QWP/1401/98 rev. 1/corr **) (2010). Available online at: https://www.ema.europa.eu/en/documents/scientific-guideline/guideline-investigation-bioequivalence-rev1_en.pdf (Accessed on December 22, 2023).

[27] ASEAN. Guideline for the conduct of bioequivalence studies. Singapore: Vientiane, Lao PDR (2015). Available online at: https://asean.org/wp-content/uploads/2021/01/ASEAN-Guideline-for-the-Conduct-of-Bioavailability-and-Bioequivalence-Studies.pdf (Accessed February 11, 2024).

[28] Chinese Taipei Food and Drug Administration. Ministry of Health and Welfare. Regulations for registration of medicinal products appendix 4 (2023). Available online at: https://law.moj.gov.tw/ENG/LawClass/LawGetFile.ashx?FileId=0000007997&lan=E (Accessed on December 27, 2023).

[29] Chinese Taipei Food and Drug Administration. Ministry of Health and Welfare. Regulations of bioavailability and bioequivalence studies (article 8.5) (2023). Available online at: https://law.moj.gov.tw/ENG/LawClass/LawAll.aspx?pcode=L0030065 (Accessed on December 27, 2023).

[30] Chinese Taipei Food and Drug Administration. Ministry of Health and Welfare. Guidelines for the verification of therapeutic equivalence of topical preparations for skin application (2023). Available online at: https://www.fda.gov.tw/tc/includes/GetFile.ashx?id=f638097323428857634 (Accessed on December 22, 2023).

[31] Ministry of Food and Drug Safety. The Republic of Korea. Regulation for pharmaceutical approvals, notifications and reviews. Notification no. 2021-90. Partially amended and enforced on Nov 11, 2021 (2023). Available online at: https://www.mfds.go.kr/eng/brd/m_18/down.do?brd_id=eng0003&seq=71524&data_tp=A&file_seq=1 (Accessed on December 29, 2023).

[32] Ministry of Food and Drug Safety. The Republic of Korea. Regulation for pharmaceutical approvals, notifications and reviews. Notification no. 2023-90. Partially amended and enforced on Dec 27, 2023 (2023). Available online at: https://www.mfds.go.kr/brd/m_207/view.do?seq=14944&srchFr=&srchTo=&srchWord=%ED%97%88%EA%B0%80&srchTp=0&itm_seq_1=0&itm_seq_2=0&multi_itm_seq=0&company_cd=&company_nm=&Data_stts_gubun=C9999&page=1 (Accessed on January 8, 2024).

[33] Health Canada. Guidance for industry: pharmaceutical quality of aqueous solutions (2005). Available online at: https://www.canada.ca/content/dam/hc-sc/migration/hc-sc/dhp-mps/alt_formats/hpfb-dgpsa/pdf/prodpharma/aqueous_aqueuses-eng.pdf (Accessed on December 22, 2023).

[34] U.S. Department of Health and Human Services. Food and Drug Administration. Center for drug evaluation and research (CDER). Draft guidance on diclofenac sodium. Recommended July 2017 (2023). Available online at: https://www.accessdata.fda.gov/drugsatfda_docs/psg/Diclofenac%20sodium_topical%20solution_NDA%20204623_RC05-17.pdf (Accessed on December 22, 2023).

[35] U.S. Department of Health and Human Services. Food and Drug Administration. Center for drug evaluation and research (CDER). Draft guidance on diclofenac sodium. Recommended Apr 2011; revised Jun 2011 (2023). Available online at: https://www.accessdata.fda.gov/drugsatfda_docs/psg/Diclofenac_%20Sodium_sol_top_20947_RC04-11.pdf (Accessed on December 22, 2023).

[36] U.S. Department of Health and Human Services. Food and Drug Administration. Center for drug evaluation and research (CDER). ANDA submissions – refuse-to-receive standards. Guidance for Industry (2016). Available online at: https://www.fda.gov/media/86660/download (Accessed on December 24, 2023).

[37] Centre for Research on Complex Generics. Characterization of complex excipients and formulations (2023). Available online at: https://www.complexgenerics.org/education-training/characterization-of-complex-excipients-formulations/ (Accessed on January 26, 2024).

[38] U.S. Department of Health and Human Services. Food and Drug Administration. Center for Drug Evaluation and Research (CDER). Considerations for waiver requests for pH adjusters in generic drug products intended for parenteral. Ophthalmic, or Otic Use Guidance for Industry (2022). Available online at: https://www.fda.gov/media/157655/download (Accessed on January 26, 2024).

[39] U.S. Department of Health and Human Services. Food and Drug Administration. Center for drug evaluation and research (CDER). Draft guidance on acyclovir (2022). Available online at: https://www.accessdata.fda.gov/drugsatfda_docs/psg/PSG_021478.pdf (Accessed December 24, 2023).

[40] U.S. Department of Health and Human Services. Food and Drug Administration. Center for drug evaluation and research (CDER). Draft guidance on eficonazol. United States: Recommended Sept (2018). Available online at: https://www.accessdata.fda.gov/drugsatfda_docs/psg/Efinaconazole_draft_Topical%20solution_RLD%20203567_RC09-18.pdf (Accessed: December 26, 2023).

[41] U.S. Department of Health and Human Services. Food and Drug Administration. Center for drug evaluation and research (CDER). Draft guidance on betamethasone dipropionate; calcipotriene (2022). Available online at: https://www.accessdata.fda.gov/drugsatfda_docs/psg/PSG_022185.pdf (Accessed December 26, 2023).

[42] U.S. Department of Health and Human Services. Food and Drug Administration. Center for drug evaluation and research (CDER). Draft guidance on erythromycin (2023). Available online at: https://www.accessdata.fda.gov/drugsatfda_docs/psg/PSG_050617.pdf (Accessed December 26, 2023).

[43] U.S. Department of Health and Human Services. Food and Drug Administration. Center for drug evaluation and research (CDER). Draft guidance on mechlorethamine hydrochloride (2023). Available online at: https://www.accessdata.fda.gov/drugsatfda_docs/psg/PSG_202317.pdf (Accessed December 26, 2023).

[44] U.S. Department of Health and Human Services. Food and Drug Administration. Center for drug evaluation and research (CDER). Draft guidance on fluocinolone acetonide. Available online at: https://www.accessdata.fda.gov/drugsatfda_docs/psg/PSG_012787.pdf (Accessed December 26, 2023).

[45] U.S. Department of Health and Human Services. Food and Drug Administration. Center for drug evaluation and research (CDER). Draft guidance on clindamycin (2023). Available online at: https://www.accessdata.fda.gov/drugsatfda_docs/psg/PSG_050600.pdf (Accessed December 26, 2023).

[46] Ministry of Health, Labour and Welfare (MHLW), Japan. Basic principles on the bioequivalence evaluation for the generic ophthalmic aqueous solutions (2016). Available online at: https://www.nihs.go.jp/drug/be-guide(e)/Ophthalmic%20dosage%20forms.pdf (Accessed on December 28, 2023).

[47] U.S. Department of Health and Human Services. Food and Drug Administration. Center for drug evaluation and research (CDER). Draft guidance on loteprednol etabonate (2022). Available online at: https://www.accessdata.fda.gov/drugsatfda_docs/psg/PSG_210933.pdf (Accessed December 29, 2023).

[48] U.S. Department of Health and Human Services. Food and Drug Administration. Center for drug evaluation and research (CDER). Draft guidance on fluorometholone (2020). Available online at: https://www.accessdata.fda.gov/drugsatfda_docs/psg/PSG_019216.pdf (Accessed December 29, 2023).

[49] U.S. Department of Health and Human Services. Food and Drug Administration. Center for drug evaluation and research (CDER). Draft guidance on cyclosporine (2022). Available online at: https://www.accessdata.fda.gov/drugsatfda_docs/psg/PSG_214965.pdf (Accessed December 29, 2023).

[50] U.S. Department of Health and Human Services. Food and Drug Administration. Center for drug evaluation and research (CDER). Draft guidance on difluprednate (2017). Available online at: https://www.accessdata.fda.gov/drugsatfda_docs/psg/Difluprednate_ophthalmic%20emulsion_RLD%20022212_RV02-17.pdf (Accessed December 29, 2023).

[51] U.S. Department of Health and Human Services. Food and Drug Administration. Center for drug evaluation and research (CDER). Draft guidance on mesalamine (2008). Available online at: https://www.accessdata.fda.gov/drugsatfda_docs/psg/Mesalamine_draft_Rectal%20enema_RLD%2019618_RC01-08.pdf (Accessed March 19, 2024).

[52] Saudi Food and Drug Authority (SFDA). The guidelines for bioequivalence (version 3.1) (2023). Available online at: https://www.sfda.gov.sa/sites/default/files/2022-08/GCC_Guidelines_Bioequivalence31_0.pdf (Accessed on December 29, 2023).

[53] Committee for Medicinal Products for Human Use (CHMP). Guideline on equivalence studies for the demonstration of therapeutic equivalence for locally applied, locally acting products in the gastrointestinal tract (CPMP/EWP/239/95, 2018, rev. 1, Corr.1*) (2018). Available online at: https://www.ema.europa.eu/en/documents/scientific-guideline/guideline-equivalence-studies-demonstration-therapeutic-equivalence-locally-applied-locally-acting-products-gastrointestinal-tract-revision-1_en.pdf (Accessed on December 29, 2023).

[54] Chinese Taipei Food and Drug Administration. Ministry of Health and Welfare. Guidelines for the verification of therapeutic equivalence for locally acting gastrointestinal preparations (2023). Available online at: https://www.fda.gov.tw/tc/includes/GetFile.ashx?id=f638089402016192890 (Accessed on December 30, 2023).

[55] U.S. Department of Health and Human Services. Food and Drug Administration. Center for drug evaluation and research (CDER). Draft guidance on nitroglycerine (2018). Available online at: https://www.accessdata.fda.gov/drugsatfda_docs/psg/PSG_021359.pdf (Accessed on December 30, 2023).

[56] WHO Prequalification Team – Medicines (PQT/MED). Notes on the design of bioequivalence study: misoprostol (2023). Available online at: https://extranet.who.int/prequal/sites/default/files/document_files/BE_Misoprostol_November2022.pdf (Accessed on December 31, 2023).

